# The role of oral magnesium supplements for the management of stable bronchial asthma: a systematic review and meta-analysis

**DOI:** 10.1038/s41533-019-0116-z

**Published:** 2019-02-18

**Authors:** Faisal Abuabat, Abdulaziz AlAlwan, Emad Masuadi, Mohammad Hassan Murad, Hamdan Al Jahdali, Mazen Saleh Ferwana

**Affiliations:** 1 Family Medicine Residency Program, King Abdulaziz Medical City, National Guard Health Affairs, King Abdullah International Medical Research Center, King Saud bin Abdulaziz University for Health Sciences, Riyadh, Saudi Arabia; 2 Department of Medical Education, King Saud bin Abdulaziz University for Health Sciences, King Abdullah International Medical Research Center, King Abdulaziz Medical City, National Guard Health Affairs, Riyadh, Saudi Arabia; 3 Evidence-Based Practice Center, Mayo Clinic, Rochester, Minnesota USA; 4 King Saud bin Abdulaziz University for Health Sciences, King Adulaziz Medical City, National Guard Health Affairs, King Abdullah International Medical Research Center, Riyadh, 11665 Saudi Arabia; 5 King Saud bin Abdul-Aziz University for Health Sciences; National & Gulf Center for Evidence Based Health Practice, King Abdullah International Medical Research Center, Family Medicine & Primary Healthcare Department, King Abdul-Aziz Medical City, Ministry of National Guard – Health Affairs, Riyadh, Saudi Arabia

## Abstract

Asthma is a chronic lung disease characterized by airway inflammation and hyper-responsiveness of airway smooth muscles. There is growing evidence that magnesium may have a role in managing asthma through its dual effect as an anti-inflammatory and bronchodilating agent. To assess the efficacy of oral magnesium supplements in chronic asthmatic patients. In addition to searching through Clinicaltrials.gov/ and references for oral magnesium supplement studies, we performed a database search in Medline, CINAHL, CENTRAL, and Embase. We contacted the authors of the included trials to ask for additional information. We included randomized controlled trials that compared oral magnesium supplements versus placebo, in addition to standard asthma treatment in mild-moderate asthmatic adults and children (older than 6 years). Two reviewers independently performed the study selection, data abstraction, and the assessment of the risk of bias. Eight trials at moderate risk of bias enrolling a total of 917 patients were included. Oral magnesium improved FEV1 at week 8 (5.69 (L/min); 95% CI: 1.92, 9.46; *I*^2^: 45%). There was no significant improvement in FEV1 at other follow up periods. There was no significant change in FVC, Methacholine challenge test, the frequency of bronchodilator use, or symptoms score. There were no data on mortality or quality of life. Oral magnesium supplements may lead to improvement in FEV1 that was only demonstrated at eight weeks; but no effect on any other outcome. Until future evidence emerges, oral magnesium cannot be recommended as adjuvants to standard treatment for mild to moderate asthmatic individuals.

## Introduction

Asthma is a chronic inflammatory disease that affects ~300 million people worldwide,^1^ reaching up to 18% of the population in certain countries.^1,2^

Symptoms of asthma include coughing, wheezing, chest tightness, and other respiratory complaints. Most of the time these symptoms are mild and can be controlled with inhalers and avoidance of known allergens, but other times they can lead to exacerbations that can be life threatening. An estimated annual 300,000 deaths worldwide are attributed to asthma.^3,4^

The mainstay of management is a beta-agonist and corticosteroids inhaler. However, new investigations into the pathogenesis of the disease are emerging. For example, many reports have observed that low blood levels and low dietary intake of magnesium are factors that possibly contribute to the development of asthma.^5–9^ In addition, low levels of magnesium have been detected in asthmatics compared with non-asthmatics, especially those that have presented to the emergency department with exacerbations.^8–12^

Magnesium deficiency has a role in many diseases in addition to asthma, including migraines, depression, and epilepsy.^6,7,13–15^ Although the exact role magnesium has in asthma is not completely understood, it is known that it functions as an anti-inflammatory agent in addition to its role in inhibiting the effect of calcium to contract smooth muscle.^6,16–19^

Previous trials showed that magnesium use through intravenous or inhaled routes does have a role in managing asthma in acute exacerbations.^20–31^ However, studies published on the benefits of oral supplements have reported unclear conclusions.^7,32–36^ Major problems with these trials regarding randomization, placebo use, intervention duration, outcome measurements, and baseline level of serum magnesium may explain the conflicting results.

The role of intravenous and inhaled magnesium in the management of acute asthma has been extensively studied and the guidelines are well established.^37,38^ IV or inhaled routes can be used as a last resort for severe, persistent asthma that fails to respond to conventional treatment.^39,40^ Nevertheless, oral magnesium is not included in these guidelines.

Our aim in this study was to conduct a meta-analysis of all published reports on the effect of oral magnesium for the management of chronic asthma.

## Results

### Search results

**Fig. 1:**
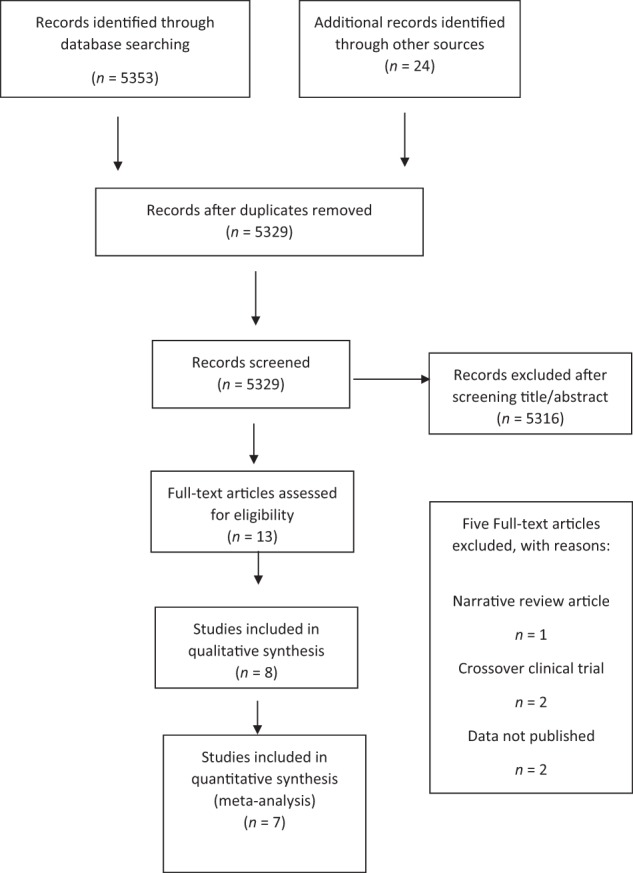
PRISMA study selection flowchart

A total of 5353 citations were identified by searching the databases; a manual search of the references and ongoing trials revealed an additional 24 articles. After screening the titles and abstracts and removing duplicates, 13 trials met our criteria. Full articles were retrieved and a further five articles were excluded, including crossover trials,^33,41^ on-going RCTs with no published results,^4,42^ and a narrative review.^43^ A total of eight trials were included in the present report.^32,34–36,44–47^ Figure 1 shows a flowchart depicting the process of selection and exclusion.^48^

### Study description

Of the eight enrolled articles, six were in English, one in Persian, and one in Russian.^45,47^ The trials were conducted in, Hungary, UK, USA, Brazil, Iran, and Russia. The total population was 917; four trials included adults and children, the other four only children, with age groups ranging from 4 to 60 years old. The enrolled subjects were labeled with either mild or moderate asthma. Type and dose of magnesium supplements were different across each trial; 200–290 mg Mg-citrate was used in three RCTs,^44–46^ two trials used 340 mg Mg-citrate,^36,47^ 450 mg Mg amino-chelate was utilized in two trials,^32,35^ and one study chose 300 mg Mg-glycine.^34^ Durations of treatment were 4–26 weeks. Measured outcomes included changes in FEV1^32,34,36,44,46,47^ and FVC^32,34,36,47^, frequency of bronchodilator use,^32,34,44–46^ asthma symptom scores,^32,44–46^ and methacholine challenge test.^32,34,36^ A summary of the characteristics is provided in Table 1.

**Table 1:** Evidence table: study description and characteristics

S. no	Setting	Study type	Age/gender	Duration	Intervention group		Control group	Outcomes
					Sample size, exposure and dose	Intervention and dose	Sample size, control and dose	
1. Bede et al.^46^	Outpatient setting, Hungary	Randomized, double-blind, placebo-controlled trial	Children aged 4–16 years. 24 boys and 16 girls (17 M/7F) in the intervention group (11 M/5F) in the placebo group	4-weeks run-in period. 12-week treatment period.	24 children received the intervention	16 children, children < 7 years old received 200 mg, and children > 7 years old received 290 mg Mg-citrate daily	16 children received 260 mg glucose placebo tablets	Changes in FEV1, bronchodilators dose and daytime symptoms scores were recorded on a personal diary card.
2. Bede et al.^44^	Outpatient setting, Hungary	Randomized, double‐blind, placebo‐controlled trial	Aged 4–16 years. 62 boys and 27 girls. (40 M/14F) in the intervention group, and (22 M/13F) in the placebo group	4-week run-in period, 12-week treatment period	54 patients received the intervention.	Children < 7 years old received 200 mg, and children > 7 years old received 290 mg Mg-citrate daily	35 patients received 260 mg glucose as a placebo	The number of bronchodilator doses and daytime activity and symptoms, night-time awakenings, scores were recorded each day on a personal diary card, measurement of FEV1 at each visit was chosen to follow up progression
3. Fogarty et al.^32^	24 primary care practices, Nottingham UK	Randomized, placebo-controlled, double-blind parallel-group trial	Aged 18–60 years. 112 males and 205 females (33 M/66F) in the Mg intervention group (42 M/64F) in the placebo group	3-week run-in period, 16-week treatment period	99 patients	Magnesium amino-chelate 450 mg/day (27.6 mmol) plus vitamin C placebo	106 patients double-matched placebo	Primary outcome measure was change between weeks 0 and 16 in a single summary statistic, which combined categorical changes in all of the below measures, with equal weight given to each outcome (−1 for worse, 0 for same and +1 for better asthma control) Secondary outcomes: FEV1, FVC, average morning and evening peak flow, average daily bronchodilator use and daily symptom score recorded in a diary for the preceding 2 weeks. (Inhaled dose of methacholine causing a 20% fall in FEV1 (PD20). to a maximum dose of 12.25 mmol methacholine)
4. Fogarty et al.^35^	24 primary care practices, Nottingham UK	Randomized, placebo-controlled, double-blind parallel-group trial	Aged 18–60 years. 40 males and 52 females (8 M/31F) in the Mg intervention group (18 M/14F) in the placebo group	10 weeks	31 patients	Magnesium amino-chelate 450 mg/day (27.6 mmol) plus vitamin C placebo	32 patients double-matched placebo	Decrease in individual inhaled corticosteroid dose achieved in each active supplement group relative to placebo
5. Kazaks et al.^36^	Outpatient setting, USA	Randomized, placebo-controlled, double-blind parallel-group trial	16 males and 36 females, (7M/18F) in the intervention group (9M/18F) in the placebo group	6.5 months	28 patients	340 Mg-citrate daily	27 patients placebo. (two patients were lost to follow up)	Primary outcome: change in bronchial responsiveness as measured by a positive methacholine challenge test in which methacholine provoked a 20% decrease in FEV1 from baseline Secondary outcomes: changes in subjective measures of asthma control, pulmonary function tests, indices of bronchial and systemic inflammation, and Mg status
6. Gontijo-Amaral et al.^34^	Outpatient setting, division of pulmonology, allergy and immunology, Brazil	A double-blind randomized parallel placebo-controlled study	Aged 7–19 years. 19 males and 18 females (12 M/6F) received the intervention (7 M/12F), received the placebo	2 months	18 patients	300 mg magnesium-glycine daily	19 patients received glycine as placebo	Primary outcome: bronchial reactivity evaluated with methacholine challenge test (PC20) Secondary outcome: asthma symptoms, lung function and allergen-induced skin responses
7. Fathi et al.^47^	Outpatient setting, Allergy & pulmonology department, Iran	Double-blind, placebo-controlled clinical trial	Asthmatics 19–55 years of age. 53 males and 47 females (25 M/25F) received the intervention, (28 M/22F) the placebo	2 months	50 patients	340 mg of Mg-citrate	50 patients Matched placebo	FEV1, FVC and FEV1/FVC ratio
8. Petrov et al.^45^	Outpatient setting, MOH, Russia	Open-labeled comparative randomized parallel-group study	Asthmatic children, aged 6–18 years. 36 males and 14 females (17 M/8F) received the intervention, (19 M/6F) were in the control group	24 weeks	25 patients	Magnesium B6 forte (Sanofi-Aventis, France) orally at a dose of 20 mg/kg/day (maximum dose of 2000 mg/day), they received tablets 1 month prior to the study?	25 patients No placebo is given	Daytime symptoms, night-time symptoms, bronchodilator use and number of asymptomatic days

#### Risk of bias

The Cochrane ROB assessment tool was used to evaluate the eight included trials (Supplementary Figures S1 & S2). We identified a low ROB in the assessed sequence generation in three articles,^32,35,36^ whereas the ROB was unclear in the remaining articles. Allocation concealment was unclear across all trials. Five trials displayed a low ROB regarding blinding,^32,34–36,47^ whereas the ROB was unclear in two trials,^44,46^ and high-risk in one study.^45^ No dropouts were reported in four trials,^34,44–46^ whereas the remaining trials reported dropouts with a variety of reasons provided.^32,35,36,47^ None of the articles provided their study protocol; therefore, selective outcome reporting could not be assessed.

### Outcomes

#### FVC

**Fig. 2:**
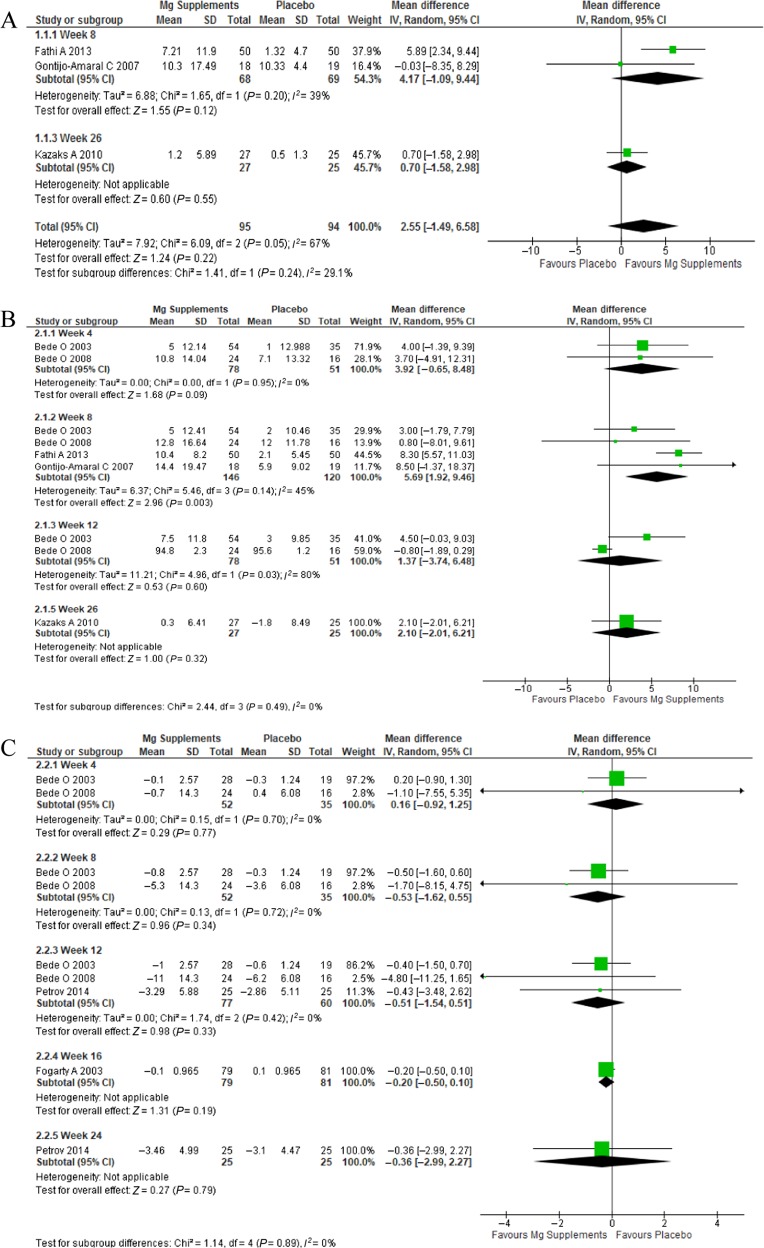
**a** FCV comparison of Mg supplements vs. placebo, **b** FEV1 comparison of Mg supplements vs. placebo, **c** bronchodilator use comparison of Mg supplements vs. placebo

Three trials reported FVC (Fig. 2a).^34,36,47^ A fourth study was excluded because it reported the change in FVC using a different unit.^32^ There was no statistically significant difference in FVC at 8 weeks, 26 weeks, or overall.

#### FEV1

Five trials reported FEV1 results (Fig. 2b).^34,36,44,46,47^ A sixth study was excluded from the analysis because it used different units.^32^ Data were available to assess the effect at weeks 4, 8, 12, and 26. The mean difference in FEV1 was only statistically significant at week 8 (5.69; 95% CI: 1.92, 9.46) with moderate heterogeneity (*I*^2^: 45%).

#### Bronchodilator use

Four trials included bronchodilator use (Fig. 2c).^32,44–46^ A fifth study was excluded due to the results that were reported as number of days rather than number of puffs as recorded in the other trials.^34^ Subgroup analysis was used according to duration at 4, 8, 12, 16, and 24 weeks. There was no statistically significant difference at any follow up period.

#### Symptoms scores

Both Bede et al.^46^ and Petrov et al.^45^ showed significant improvement in daytime symptoms after 12 weeks in the intervention group but not in other measures. Kazaks et al.^36^ on the other hand used two questionnaires (AQLQ and ACQ) to assess subjective measures, statistically significant improvements were noted after 6.5 months in the AQLQ.

#### PD20 inhaled methacholine challenge test

Three trials performed a PD20 inhaled methacholine challenge test.^32,34,36^ Subgroup analysis was used at weeks 8, 16, and 24 (Supplementary Figure S3). The mean difference was not significant at week 8 but reached statistical significance at week 16 (−0.14; 95% CI: −0.28, −0.0).

Supplementary Table 1 presents GRADE summary of finding for all outcomes.

#### Quality of evidence

The quality of evidence (i.e., certainty in the estimates) was low, reduced because of risk of bias (lack of clear allocation concealment) and imprecision (mostly nonsignificant results and small sample size). The outcomes available in the trials were surrogate outcomes except for symptoms score and possibly bronchodilators use.

## Discussion

This systematic review and meta-analysis was performed to evaluate the effects of oral magnesium on chronic asthma. The results of the measured outcomes was overall precise and nonsignificant (except for FEV1 at week 8). None of the trials reported on mortality or adverse events. Certainty in these estimates is low. There was some high heterogeneity, which could be explained by different type, dose, or duration of the intervention across the experimental groups in the involved RCTs. We excluded crossover RCTs due to uncertainty over washout periods of magnesium. We have identified ongoing trials registered in Clinicaltrials.gov, which might have future implications on the conclusion reached in this study.^4,42^

The role of magnesium in bronchial asthma has been discussed in the recent GINA guidelines. The evidence supports the use of intravenous magnesium in acute situations, especially with asthmatics that do not respond to initial management. However, the routine use of intravenous magnesium in acute or chronic asthma is not supported by evidence.^49^ Nevertheless, the use of oral magnesium for the prevention of asthma exacerbations or to improve control has not been discussed in the GINA report. Other supplements, such as vitamin D, were mentioned in GINA.^4^ In the present study, only FEV1 was shown to be significantly improved with oral magnesium.

However, encouraging findings were found especially in Kazak’s study including a significant increase in the concentration of methacholine required to cause a 20% drop in FEV1 at 6 months, and a 5.8% improvement in PEFR and an improvement in subjective measures (AQLQ, ACQ) all noticed at 6.5 months in subjects who were in the treatment group. Therefore, there is a need for future large, high quality RCTs to reliably determine the role of oral magnesium in asthma management. To our knowledge, this is the first systematic review and meta-analyses evaluating the use of magnesium supplements as an adjunct treatment to inhalers for asthmatics.

There are several limitations to this study. First, sample sizes are considered relatively small for a common disease like asthma, some trials recruited less than fifty participants.^34,44,45^ Second, important data were missing in some of the trials; strategies we used to overcome this problem were discussed in the methodology above. Additionally, we were not able to assess the effect of the intervention on reducing the dose of inhaled steroid, because it was only reported in a single study which showed no benefit.^35^

Furthermore, GINA guidelines defined adults as those 6 years old and above. This dichotomy is not used in many of the available studies. Clearer details regarding trial methodology, particularly about sequence generation and allocation concealment would help future systematic reviewers appraise the literature more properly. Additionally, clear stratification by oral magnesium dose and duration of therapy can help produce more precise and useful estimates. Finally, none of the RCTs provided their research protocol.

The use of magnesium supplements as an adjuvant to standard asthma treatment in mild to moderate asthmatic patients is not supported by high quality evidence. Apart from one point at week 8 where the FEV1 had significantly improved, no other benefits from oral magnesium supplements were seen. Until future higher quality evidence emerges, oral magnesium cannot be recommended for mild to moderate asthma.

## Methods

### Search strategy

A medical librarian (MVN) performed a comprehensive literature search on April 2016. Searched databases were as follows: Medline, CINAHL, Cochrane Central Register of Controlled Trials (CENTRAL), Embase, and on-going clinical trials (https://clinicaltrials.gov/). The main search concepts were asthma and oral magnesium; Boolean operators: OR (for combining synonyms), AND (to combine concepts), and truncations were used when appropriate. A review of references and a manual search of relevant articles was completed. Additionally, we contacted the authors of the trials to minimize chances of missed articles. The search was updated on May 2017. No restrictions to study type, time, or language were applied.

### Inclusion and exclusion criteria

We included RCTs that investigated the use of oral magnesium as an adjunct to asthma inhalers. Asthma was defined in accordance with the authors of the included trials, which were either clinically, objectively (spirometry), or both. All trials fulfilled the inclusion criteria as follows: (1) Population: mild and moderate asthmatic older than 6 years [6 years was the cut-off based on the global initiative for asthma (GINA) guidelines];^4^ (2) Intervention: all preparations of oral magnesium, regardless of name or dosage; (3) Comparisons: placebo or no treatment; (4) Outcomes: frequency of rescue asthma exacerbation, ER visits or hospital admissions, objective measures: FEV1, FVC, and PEF, daytime symptoms, daytime activity, night-time symptoms, frequency of bronchodilator use, use of oral or inhaled steroids, side effects, and mortality; (5) Timing: treatment for any duration was included; (6) Study type: RCTs, blinded or open-labeled. Crossover trials were excluded.

### Study selection

Two reviewers (AA and FA) separately scanned the titles and abstracts of all articles, and when needed, the full text was retrieved and reviewed to identify all relevant articles. A third author (MF) resolved any disagreements.

### Data extraction

Important data were extracted using a piloted form, which incorporated all attributes of the studies, including author, publication year, country, setting, design, sample size, intervention (dose and type), placebo, patient’s age and sex, severity, study duration, inclusion and exclusion criteria, outcomes, and limitations. Data was extracted by AA and FA and reviewed by MF. We contacted the author of the study in question for any missing information.

### Methodological quality assessment

The risk of bias (ROB) was assessed using the Cochrane ROB assessment tool;^38^ this was done independently by AA and FA and disagreements were resolved by MF.

### Statistical analysis

We combined the results using RevMan 5.3.5. There were five outcomes, which included FVC, FEV1, methacholine challenge test, bronchodilator use, and symptoms score. All outcomes were considered continuous data; therefore, we used the weighted mean difference (WMD).^44,45^ A random-effects model was used to pool the results because of anticipated high heterogeneity due to the different characteristics and qualities of the included studies. Subgroup analysis was used according to the duration of the intervention. Subgrouping was not performed based on severity because the included RCTs did not separate data based on severity except for one,^44^ which data were combined. To assess for heterogeneity, we used the *I*^2^ level of 50% or above, and Cochrane *Q* test which was considered significant if the *p*-value < 0.05.

Symptoms were considered an important clinical outcome. Symptom scores were reported differently in each trial, therefore it was not feasible to combine across trials in meta-analysis. Therefore, symptom scores were reported narratively and separately in the results section.

Four RCTs were available for analysis.^32,44–46^

### Missing data

The standard deviations (SD) of the mean difference before and after treatment was missing in select trials. Therefore, we contacted the authors of each study and received no response, thus we recalculated the data whenever the *p*-value was available, and when neither the *p*-value nor SD was available, we used the SD of similar studies.^44,46^ In addition, the FEV1 and FVC units were not defined in one study, we contacted the author with no response, and thus we excluded these outcomes.^32^ The present study was approved by King Abdullah International Medical Research Center IRB committee on November 15, 2016 (research number RC16/084/R).

## Supplementary information

Supplementary Information

## Supplementary information

**Supplementary information** accompanies the paper on the *npj Primary Care Respiratory Medicine* website (10.1038/s41533-019-0116-z).
