# Supplementary material for: The role of oral magnesium supplements for the management of stable bronchial asthma: a systematic review and meta-analysis
Source: NPJ Prim Care Respir Med. 2019 Feb 18;29:4. doi: 10.1038/s41533-019-0116-z (PMC6379356; doi:10.1038/s41533-019-0116-z)
Supplement: Supplementary file 1 — Supplementary Information [file 41533_2019_116_MOESM1_ESM.doc]

**SUPPLEMENTARY INFORMATION**

**TITLE: The role of oral magnesium supplements for the management of stable bronchial asthma: A systematic review and meta-analysis**

**Authors’ full names:** Faisal Abuabat^1,2,3^ , Abdulaziz AlAlwan^1,2,3^ , Emad Masuadi^2,3,1^ , Mohammad Hassan Murad ^4^, Hamdan Al Jahdali^1,2,3^, Mazen Saleh Ferwana^2,3,1^

**Authors’ affiliation(s):**, King Abdulaziz Medical City, National Guard Health Affairs^1^, King Abdullah International Medical Research Center^2^, King Saud bin Abdulaziz University for Health Sciences^3^, Riyadh, Saudi Arabia, (Murad MH), Evidence-based Practice Center, Mayo Clinic, Rochester, Minnesota, USA,^4^

**Figure S1** Cochrane ROB assessment tool

**
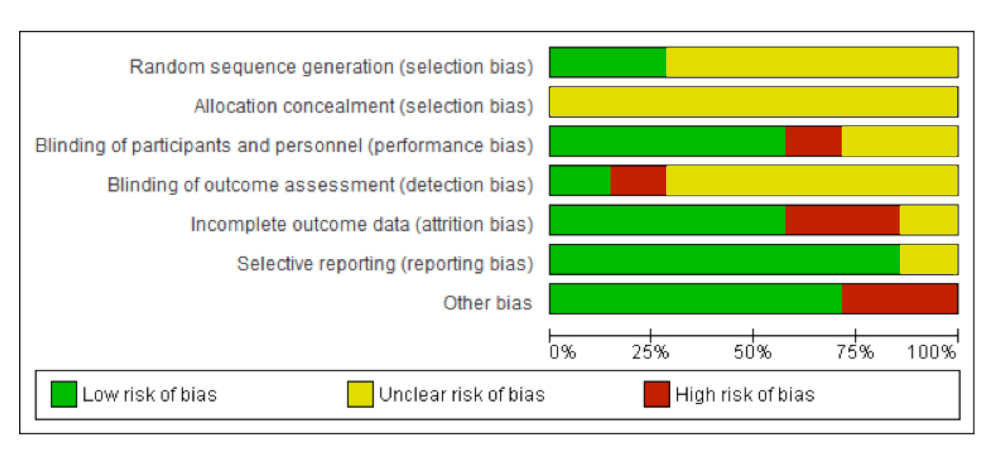
Figure S2***-* Cochrane ROB assessment too

**
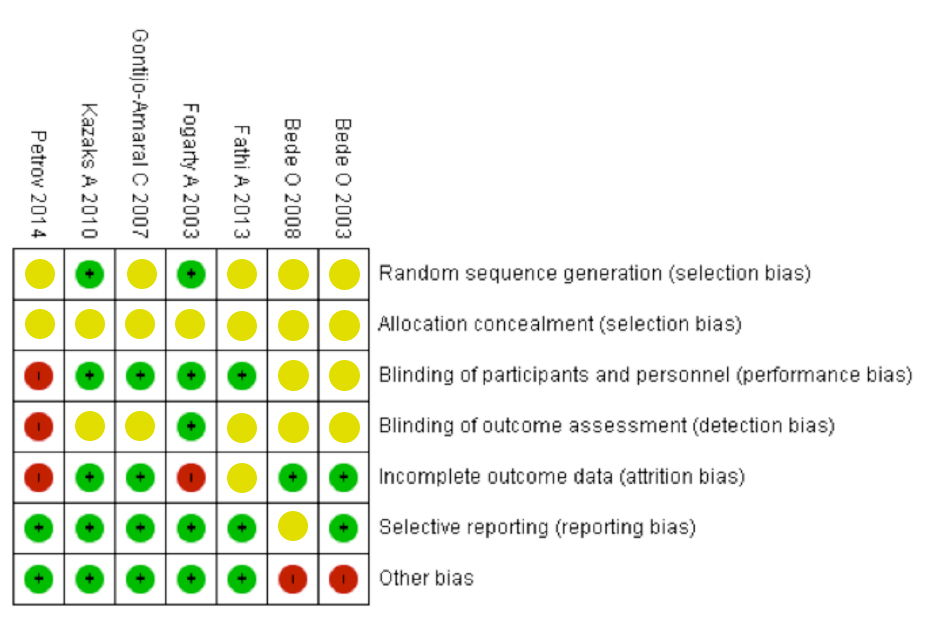
**

**Figure S3 PD20 Inhaled methacholine challenge test**

**
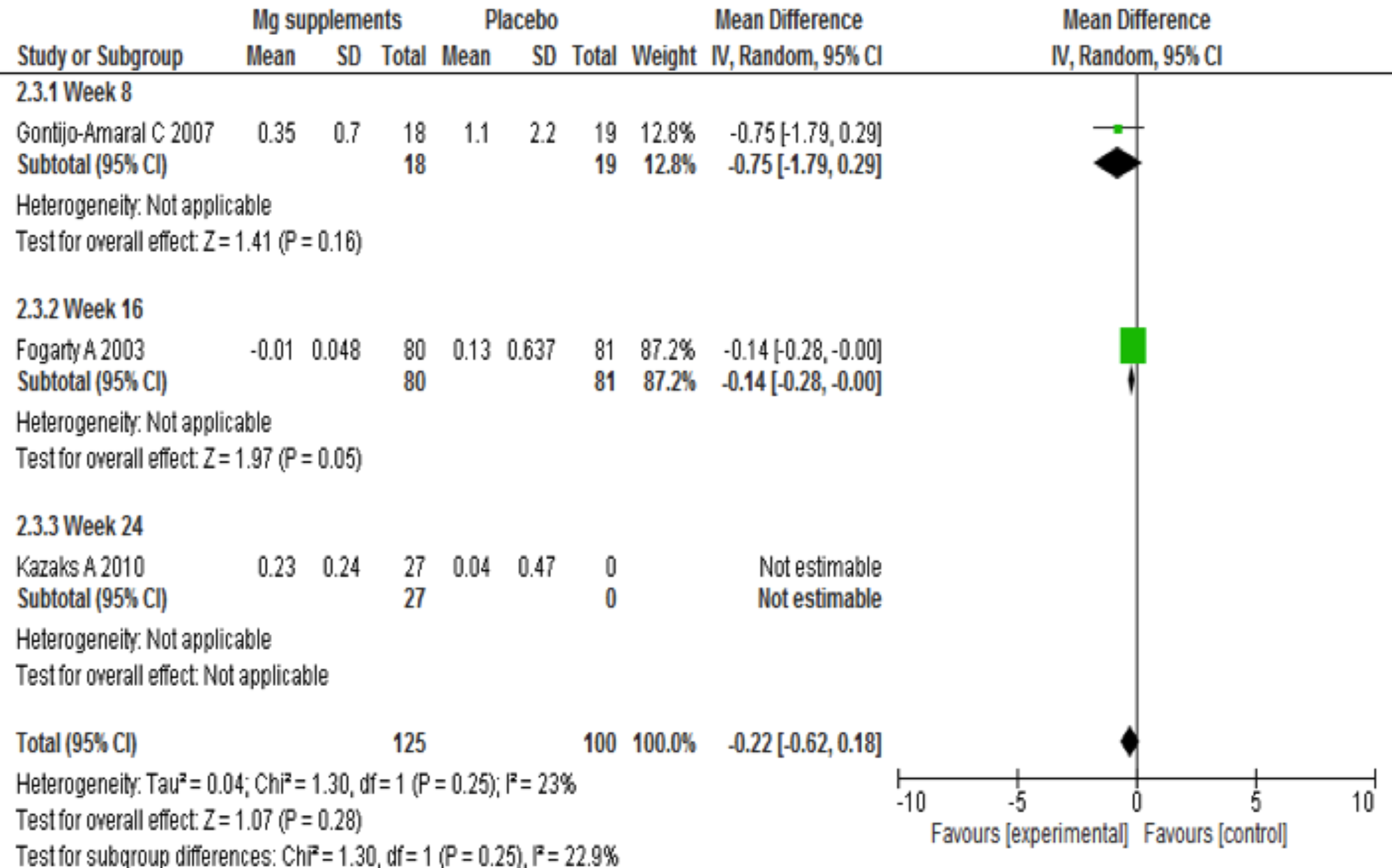
**

**Table S1-****GRADE summery of findings**

| **Mg Supplements compared to Placebo for treatment of chronic Bronchial Asthma** | | | | | | | | |
| --- | --- | --- | --- | --- | --- | --- | --- | --- |
| **Patient or population:** patients with [health problem] **Settings:**  **Intervention:** Mg Supplements **Comparison:** Placebo | | | | | | | | |
| **Outcomes** | **Illustrative comparative risks* (95% CI)** | | | **Relative effect (95% CI)** | | **No of Participants (studies)** | **Quality of the evidence (GRADE)** | **Comments** |
|  | Assumed risk | Corresponding risk | |  |  |  |  |  |
|  | **Placebo** | **Mg Supplements** | |  | |  |  |  |
| FVC |  | The mean FVC in the intervention groups was **2.55 higher** (1.49 lower to 6.58 higher) | |  | | 189 (3 studies) | ⊕⊝⊝⊝ **very low**^1,2,3,4^ |  |
| FVC **- Week 8** |  | The mean FVC - week 8 in the intervention groups was **4.17 higher** (1.09 lower to 9.44 higher) | |  | | 137 (2 studies) | ⊕⊝⊝⊝ **very low**^5,6,7^ |  |
| FVC **- Week 26** |  | The mean FVC - week 26 in the intervention groups was **0.7 higher** (1.58 lower to 2.98 higher) | |  | | 52 (1 study) | ⊕⊝⊝⊝ **very low**^8,9,10^ |  |
| *The basis for the **assumed risk** (e.g. the median control group risk across studies) is provided in footnotes. The **corresponding risk** (and its 95% confidence interval) is based on the assumed risk in the comparison group and the **relative effect** of the intervention (and its 95% CI).  **CI:** Confidence interval; | | | | | | | | |
| GRADE Working Group grades of evidence **High quality:** Further research is very unlikely to change our confidence in the estimate of effect.  **Moderate quality:** Further research is likely to have an important impact on our confidence in the estimate of effect and may change the estimate. **Low quality:** Further research is very likely to have an important impact on our confidence in the estimate of effect and is likely to change the estimate. **Very low quality:** We are very uncertain about the estimate. | | | | | | | | |
| ^1^ Randomization is unclear in 2 studies (Fathi and Contijo), Concealment is unclear in the 3 studies, and blinding is unclear in all studies ^2^ heterogeneity, I sq is 67% ^3^ surrogate outcome ^4^ OIS not met because of small sample size, and 95% CI crosses the clinical threshold ^5^ OIS not met because of small sample size, and 95% CI crosses the clinical threshold ^6^ Concealment and blinding are unclear  ^7^ surrogate outcome ^8^ Concealment and blinding are unclear  ^9^ surrogate outcome ^10^ OIS not met because of small sample size, and 95% CI crosses the clinical threshold | | | | | | | | |
| Mg Supplements compared to Placebo for [health problem] | | | | | | | | |
| Patient or population: patients with [health problem] Settings:  Intervention: Mg Supplements Comparison: Placebo | | | | | | | | |
| **Outcomes** | **Illustrative comparative risks* (95% CI)** | | **Relative effect (95% CI)** | **No of Participants (studies)** | **Quality of the evidence (GRADE)** | **Comments** |  |  |
|  | Assumed risk | Corresponding risk |  |  |  |  |  |  |
|  | **Placebo** | **Mg Supplements** |  |  |  |  |  |  |
| **FEV1 - Week 4** |  | The mean fev1 - week 4 in the intervention groups was **3.92 higher** (0.65 lower to 8.48 higher) |  | 129 (2 studies) | ⊕⊝⊝⊝ **very low**^1,2,3^ |  |  |  |
| **FEV1 - Week 8** |  | The mean fev1 - week 8 in the intervention groups was **5.69 higher** (1.92 to 9.46 higher) |  | 266 (4 studies) | ⊕⊕⊝⊝ **low**^4,5^ |  |  |  |
| **FEV1 - Week 12** |  | The mean fev1 - week 12 in the intervention groups was **1.37 higher** (3.74 lower to 6.48 higher) |  | 129 (2 studies) | ⊕⊝⊝⊝ **very low**^6,7,8^ |  |  |  |
| **FEV1 - Week 26** |  | The mean fev1 - week 26 in the intervention groups was **2.1 higher** (2.01 lower to 6.21 higher) |  | 52 (1 study) | ⊕⊝⊝⊝ **very low**^9,10,11^ |  |  |  |
| **Bronchodilator use - Week 4** |  | The mean bronchodilator use - week 4 in the intervention groups was **0.16 higher** (0.92 lower to 1.25 higher) |  | 87 (2 studies) | ⊕⊕⊕⊝ **moderate**^12^ |  |  |  |
| **Bronchodilator use - Week 8** |  | The mean bronchodilator use - week 8 in the intervention groups was **0.53 lower** (1.62 lower to 0.55 higher) |  | 87 (2 studies) | ⊕⊕⊕⊝ **moderate**^13^ |  |  |  |
| **Bronchodilator use - Week 12** |  | The mean bronchodilator use - week 12 in the intervention groups was **0.51 lower** (1.54 lower to 0.51 higher) |  | 137 (3 studies) | ⊕⊕⊝⊝ **low**^14,15^ |  |  |  |
| **Bronchodilator use - Week 16** |  | The mean bronchodilator use - week 16 in the intervention groups was **0.2 lower** (0.5 lower to 0.1 higher) |  | 160 (1 study) | ⊕⊕⊝⊝ **low**^16,17^ |  |  |  |
| **Bronchodilator use - Week 24** |  | The mean bronchodilator use - week 24 in the intervention groups was **0.36 lower** (2.99 lower to 2.27 higher) |  | 50 (1 study) | ⊕⊕⊝⊝ **low**^18^ |  |  |  |
| **PD20 Inhaled Methacholine Challenge Test** |  | The mean pd20 inhaled methacholine challenge test in the intervention groups was **0.22 lower** (0.62 lower to 0.18 higher) |  | 225 (3) |  |  |  |  |
| **PD20 Inhaled Methacholine Challenge Test - Week 8** |  | The mean pd20 inhaled methacholine challenge test - week 8 in the intervention groups was **0.75 lower** (1.79 lower to 0.29 higher) |  | 37 (1) | See comment |  |  |  |
| **PD20 Inhaled Methacholine Challenge Test - Week 16** |  | The mean pd20 inhaled methacholine challenge test - week 16 in the intervention groups was **0.14 lower** (0.28 lower to 0 higher) |  | 161 (1) | See comment |  |  |  |
| **PD20 Inhaled Methacholine Challenge Test - Week 24** | See comment | See comment | Not estimable | 27 (1) | See comment |  |  |  |
| *The basis for the **assumed risk** (e.g. the median control group risk across studies) is provided in footnotes. The **corresponding risk** (and its 95% confidence interval) is based on the assumed risk in the comparison group and the **relative effect** of the intervention (and its 95% CI).  **CI:** Confidence interval; | | | | | | |  |  |
| GRADE Working Group grades of evidence **High quality:** Further research is very unlikely to change our confidence in the estimate of effect.  **Moderate quality:** Further research is likely to have an important impact on our confidence in the estimate of effect and may change the estimate. **Low quality:** Further research is very likely to have an important impact on our confidence in the estimate of effect and is likely to change the estimate. **Very low quality:** We are very uncertain about the estimate. | | | | | | |  |  |
| ^1^ Randomization, concealment, blinding are unclear in both studies. high risk of bias for other biases domain ^2^ surrogate outcome ^3^ OIS not met because of small sample size, and 95% CI crosses the clinical threshold ^4^ most domains are unclear in all studies, and in Bedoo studies, high risk of bias for other biases domain ^5^ surrogate outcome ^6^ Randomization, concealment, blinding are unclear in both studies. high risk of bias for other biases domain ^7^ surrogate outcome ^8^ OIS not met because of small sample size, and 95% CI crosses the clinical threshold ^9^ Concealment and blinding are unclear ^10^ surrogate outcome ^11^ OIS not met because of small sample size ^12^ Randomization, concealment, blinding are unclear in both studies. high risk of bias for other biases domain ^13^ Randomization, concealment, blinding are unclear in both studies. high risk of bias for other biases domain ^14^ Petrove study, has high risk of bias for blinding and incomplete dats. Randomization, concealment, blinding are unclear in Bedo studies and high risk of bias for other biases domain ^15^ OIS not met because of small sample size ^16^ Concealment unclear and incomplete data high risk of bias ^17^ OIS not met because of small sample size ^18^ OIS not met because of small sample size ^19^ Randomization, concealment, blinding are unclear in both studies. high risk of bias for other biases domain ^20^ OIS not met because of small sample size ^21^ Randomization, concealment, blinding are unclear in both studies. high risk of bias for other biases domain ^22^ OIS not met because of small sample size ^23^ Randomization, concealment, blinding are unclear in both studies. high risk of bias for other biases domain and Petrov study has high risk of bias for blinding and incomplete data ^24^ OIS not met because of small sample size ^25^ Petrov study has high risk of bias for blinding and incomplete data, and Kazaks hasunclear concealment and blinding  ^26^ OIS not met because of small sample size | | | | | | |  |  |
